# Revisiting Early Palliative Care for Patients With Hematologic Malignancies and Bone Marrow Transplant: Why the Delay?

**DOI:** 10.7759/cureus.10504

**Published:** 2020-09-17

**Authors:** Adriana Franjul Sánchez, Angelica M Fuentes Armesto, Carlo Briones Chávez, Marco Ruiz

**Affiliations:** 1 Medicine, University of Medicine and Health Sciences, Bassettere, KNA; 2 Internal Medicine, Larkin Community Hospital, Miami, USA; 3 Hematology and Oncology, Miami Cancer Institute, Miami, USA

**Keywords:** palliative care, hematologic malignancy, bone marrow transplant, quality of life

## Abstract

Palliative care has been defined as specialized care for patients facing serious illnesses. Despite advancements in the field and studies documenting the effectiveness of early palliative care (PC) interventions in seriously ill patients, the fields of hematologic malignancies and bone marrow transplant still lag behind of a comprehensive framework for early and effective interventions. The aim of this literature review is to analyze and discuss the possible barriers to care and delayed referrals for hematologic malignancies and bone marrow transplant patients. Using the EBSCO and PubMed databases, articles regarding PC among patients with hematologic malignancies and bone marrow transplant were analyzed. There are three main domains with its respective barriers in PC: physicians, patients and caregivers, and the healthcare system. Issues that were identified included the lack of knowledge and misconceptions about PC among physicians, patients, and caregivers, delayed referral of patients with hematologic malignancies, unrealistic treatment expectations, lack of communication between specialties, difficulties with appointment availability, geographical distance between clinics, and lack of insurance coverage for PC services. We suggest possible alternatives including obligatory continuing medical education (CME) credits, loan forgiveness, rotations during residency and fellowship training, use of informational videos and pamphlets to educate patients and caregivers, obligatory early consults despite prognosis, an algorithm to evaluate patient’s needs, creating a platform within electronic medical records (EMR) systems shared by specialties, and having PC service in every cancer center. Findings suggest a need for further studies aimed towards implementing solutions to increase the early referral of patients with hematologic malignancies and bone marrow transplantation (BMT) to palliative care.

## Introduction and background

Background

Hematologic malignancies are cancers that begin in blood-forming tissue such as bone marrow and/or in the cells of the immune system [1]. The types of hematologic malignancies include acute lymphoblastic leukemia (ALL), acute myeloblastic leukemia (AML), chronic lymphoblastic leukemia (CLL), chronic myelogenous leukemia (CML), myelodysplastic syndrome (MDS), lymphomas and multiple myeloma [2]. According to the Leukemia and Lymphoma Society, in 2019, an estimated 10% of the 1,762,450 newly diagnosed cancers were leukemia, lymphoma, and myeloma and an estimated 1,399,180 people living in the USA had or currently have leukemia, lymphoma or myeloma [3]. In the case of MDS, the data is more limited. Currently, the prevalence, survival, and death statistics of MDS have started to be recorded by the Surveillance, Epidemiology, and End Results Program [4]. Estimates indicate that there are approximately 14,011 reported cases per year from the period of 2011-2015, which total about 70,056 diagnoses.

Currently, one of the treatments available for hematologic malignancies is bone marrow transplantation (BMT) [5]. A BMT is a procedure that incorporates healthy blood-forming stem cells into the patient's body to replace the damaged cells or diseased bone marrow. There are three existing types of BMT: autologous in which the donor is the patient themselves, allogeneic where the donor shares the same genetic material as the patient, and umbilical cord blood transplants in which stem cells are taken from the umbilical cord and subsequently, tested, typed, counted, and frozen, and then stored until they are needed [6]. Annual estimates for allogeneic transplants surpassed 9,028 in 2018, while autologous transplants have been steadily increasing since 2000 to 14,006 and represent 60% of all transplants in the US [7]. In the case of umbilical cord blood transplants, it was reported that more than 40,000 transplants have been performed [8].

Introduction: palliative care

Palliative care (PC) emerged from the hospice movement in the 1960s, but it was not until 2006, that PC was defined as a medical specialty [9]. Given the recent introduction of this sub-specialty, its role is sometimes not well-known or understood, which leads to one of the main obstacles that the specialty is confronting. PC is composed of a team of professionals that includes physicians, nurses, social workers, pharmacists, chaplains, dieticians, physical therapists, and paralegals [10,11]. PC has been defined as specialized care for people facing any type of serious illness [12,13]. It assesses the fundamental issues that impair the lives of patients and their caregivers including symptom management, quality of life, emotional distress, spiritual well-being, and aids in the difficult process of decision making throughout the illness. It also helps in the transition of care between specialties [14]. PC should not be confused with hospice or end-of-life care as it does not require a terminal diagnosis, proximity to death, or a gruesome prognosis in order to obtain a referral [12]. In PC, the main focus is to employ a multidisciplinary approach between specialties, thereby improving the patient's quality of life throughout their disease and not only during the late stages. As reported by the World Health Organization (WHO), 40 million people require PC annually, 33% of which have cancer and PC clinicians evaluate only 14%. Currently around the world, approximately 12 million adults and 400,000 children are facing serious illnesses and would benefit from PC services [13]. Per America’s Care of Serious Illness report, as of 2019, 72% of hospitals with more than 50 beds have PC teams in their service [13]. Approximately, only 20 countries have PC services included in their healthcare system [15]. It has been demonstrated that PC helps patients and their families by improving quality of life, symptom management, and coping methods, creating better prognostic awareness, reducing costs and hospital readmissions, and even improving overall survival [16].

The purposes of this literature review are to discuss some of the fundamental issues that PC is facing by analyzing the possible reasons for the lack of early referral for patients with hematologic malignancies and BMT and to evaluate and propose measures that could potentially improve the understanding and knowledge of PC among physicians, patients, family members, and caretakers, thereby, decreasing the delay in treatment for patients with hematologic malignancies which could potentially lead to improvements in their quality of life and overall well-being.

Using the EBSCO database, a search under the keyword “palliative care” gave a result of 415,727 articles, while “palliative care and hematologic malignancies” resulted in 7,599 articles found, and “palliative care and bone marrow transplant” in 7801 articles. The articles were further narrowed to articles published in the period of 2015 to 2020. Of those articles, less than 40 focused solely on PC for patients of hematologic malignancies treated with BMT. The search using the Pubmed database using the keyword “palliative care” showed 79,045 articles, while “palliative care and hematologic malignancies” resulted in 510 articles and “palliative care and bone marrow transplant” in 269 articles.

## Review

What problems does palliative care specialty confront?

Evidence has shown that around 50-70% of adults in the US and other countries have never heard of PC, and even if they did, their knowledge about the specialty needed to be corrected [17]. One possible contributing factor to this misconception is that PC is a relatively new specialty. Thus, its role may not be well-understood. As previously mentioned, another contributing factor is that many physicians and patients confuse PC with hospice and end-of-life care. For this reason, physicians may be reluctant to refer patients to the PC team. This confusion might be due to a lack of exposure to PC during the physician's training years, including during residency and fellowship. Evidence of this issue was a study that concluded that in respect to physician training in PC, 46% of physicians reported attending lectures and CME courses in PC, 37% had no exposure or training whatsoever, 29% completed a rotation during residency or fellowship and only 1% had more than six months of formal training in this young specialty [18]. Another potential issue found in the literature was that physicians believe they have the appropriate expertise in the management of physical symptoms of hematologic diseases. Hence, they should be the ones to coordinate the care of patients across all stages of the disease [18]. El-Jawahri et al. reported that 40% of physicians endorsed a distrust in PC specialists mainly because they believed PC clinicians lack enough understanding of hematology-oncology, thereby regarding their expertise as insufficient to treat their patients [18].

Regarding knowledge about PC, the patients’ and their family members’ understanding of PC is extremely critical to the implementation of these beneficial medical services. Their familiarity with the specialty determines their approach and perspective towards PC. A survey concluded that most patients have never heard of the term palliative, which means that there is a lack of understanding rather than a fear of what the field implies [12]. The Health Information National Trends Survey cross-sectional study in 2019 demonstrated that 71% of US adults had never heard of PC [4]. LeBlanc and El-Jawahri found that once patients and family members were educated about PC, they were more interested in the specialty and wanted to try for themselves or a family member [12].

Another potential reason for the lack of referral of cancer patients is due to unrealistic expectations about the efficacy of treatments [10,14]. There are two potential explanations for this issue. Either physicians expect that patients will improve with their current treatment or they might have the misconception that patients can’t receive PC while receiving treatment. It has been observed that physicians consult PC more often in the inpatient setting. However, studies have shown patients would benefit more when early PC services are implemented in outpatient settings and in less acute conditions [19]. Ideally, PC should be offered to patients early in the course of their disease and in the setting they prefer, including their homes, skilled nursing facilities, and clinics.

Lastly, PC clinicians also identify communication as an issue mainly due to the lack of a unified electronic medical record system for sharing information between specialties. If PC specialists are unable to access their patient's information, they could miss critical details regarding changes in prognosis, treatment details, and important discussions of disease management in which they would be able to offer their expertise and provide insight not only to patients, their family members, and caregivers but also to hematologic oncologists. This lack of communication prevents optimal care of patients by impeding a collaborative interdisciplinary approach to healthcare [20].

Why are hematologic malignancy patients not referred to palliative care or referred too late?

Patients with hematologic malignancies, similar to patients with solid tumors, experience many changes during the course of their disease, including physical symptoms and psychological distress. These patients may suffer from pain, depression, anxiety, confusion, fatigue, breathlessness, insomnia, nausea, constipation, diarrhea, and anorexia. For patients of hematologic malignancies, their disease can be so distressing that, as evidenced by a prospective cohort study, 37% of patients met the criteria for sub-threshold acute stress disorder [17]. In some of these patients, their disease takes unpredictable courses, rendering their prognosis uncertain. For these reasons, patients with hematologic malignancies should be referred to PC soon after their initial diagnosis, to help them cope with and manage these dramatic changes. There is a significant gap in PC between patients with solid tumors and hematologic malignancies. LeBlanc et al. found that patients with hematologic malignancies are not referred to PC as often as patients with solid tumors [16].

Other reasons that might lead to delay in referral include the heterogeneity of hematologic malignancies, the attitudes of hematologists towards the specialty, and the misconceptions about the utility of PC [14]. A study by LeBlanc et al. compared the perceptions of hematologist oncologists and solid tumor oncologists about PC. The study showed that 61% of hematologist oncologists thought of PC as end-of-life care and as anti-ethical to cancer treatment compared to only 16% among solid tumor oncologists. A majority of hematologic oncologists believed PC was an alternative only when there were no more therapeutic options to offer patients and that it could not be used concurrently with the patient’s curative treatment [14].

Dasch et al. found that patients with hematologic malignancies received more intensive treatments and were more likely to die in an intensive care unit [11]. Additionally, El-Jawahri et al. showed that 43% of physicians reported collaborating with PC in the inpatient setting compared to 21% in outpatient clinics [18]. Taber et al. mentioned that in a cohort study performed in 2018, among 813 hematologic malignancy patients, only 33% of patients who died while hospitalized received a PC consult. 78% of those consults occurred while in the inpatient setting meaning that most patients were never evaluated by a PC specialist as an outpatient. In this same study, the authors pointed out the disparities that exist between patients with solid tumors and those with hematologic malignancies. Compared to hematologic malignancy patients, 47% of solid tumor patients received a PC consult and 50% of those took place in the inpatient setting [17]. This can be due in part to the close proximity of clinics and availability for integrated ward rounds between specialties in the inpatient setting. Increasing availability and accessibility of PC services could also help ensure that hematologic malignancy patients receive care where needed since the unpredictability of their disease may cause them to spend a significant amount of time between clinics, homes, and the inpatient setting [17,21]. Many studies suggest that patients who received an early referral to an outpatient PC clinic have more improvements in quality of care and even live longer compared to those who received a late PC consultation in the inpatient setting [10].

Another possible barrier is that due to the heterogeneity, aggressiveness, and unpredictable nature of hematologic malignancies, physicians may question the right timing for a referral to PC. Due to the strong bond that is developed between a patient and his/her hematologist-oncologist, a physician might feel some type of exclusive ownership to the care of the patient and the patient might have a high level of trust for only one doctor. This type of patient-physician relationship may lead to reluctance of the physician to refer the patients for evaluation. Patients get used to seeing their doctor more often, even weekly, fostering a stronger and tighter bond, which might also cloud the decision-making process of clinicians who already believe they can address their patient’s issues themselves or even better than PC specialists [22]. The view of solid organ tumors oncologists differs from the perception of hematologist oncologists in this matter. Solid-organ tumor oncologists more often perceive PC as helpful for their patients and describe it as a useful source of knowledge and a team that can help with the management of patients.

Physicians also cite difficulties with appointment availability and issues with healthcare coverage by insurance companies since unlike hospice care, most policies do not cover PC expenses [23]. Per America’s Care of Serious Illness Report, another important reason to invest in PC would be to incentivize hospitals by reducing costs, which is estimated at about $3,000 to $4,800 per patient depending on their diagnosis. Adding PC services to hospitals around the country could potentially save hundreds of millions per year [13]. Another obstacle to an early referral of hematologic malignancy patients to PC is due to policy issues in which a patient needs to meet excessive criteria in order to be justified for a PC consultation [20].

Why should we refer bone marrow transplant patients to palliative care?

Patients that are undergoing or have undergone the process of BMT face changes in their daily living, including the acceptance of their diagnosis, symptom burden, continuous procedures, aggressive treatments, and even post-procedure complications. A prospective study of patients undergoing BMT showed that 68% of patients reported pain, with 23% of those experiencing severe pain, while 78% experienced nausea and 89% insomnia. The study also showed that patients admitted for stem cell transplants endorsed an increase in post-transplant depression from 15.6% at baseline to 37.8% eight days post-treatment [17]. In addition, it has been reported that BMT patients usually receive the most intensive treatments during their last 30 days of life [24]. Therefore, there is even more need for early PC interventions in these populations. The early integration of PC has been proven to improve outcomes, symptom burden, quality of life, psychological symptoms, and illness understanding for patients [18]. El-Jawahri et al. analyzed the life of patients undergoing hematopoietic stem cell transplants who had a PC intervention during the hospitalization period. The study compared these patients with others undergoing the same procedures but without PC intervention and it demonstrated that patients within the intervention group benefited from a better quality of life, lower depression scores of 30.0% vs. 59.7%, and less anxiety of 10.0% vs. 41.6% with only two weeks of intervention [25].

It is essential to understand the perception of patients undergoing BMT about PC. Based on the literature review, we found a pilot study in which they implemented early integration of PC in patients undergoing stem cell transplants. Most of these patients declined to enroll in the intervention when they were approached. Their hesitance to enroll was mainly due to the title of the study having the term “palliative care”. But when the title was changed to “supportive care”, the patients were more eager to participate [5].

Discussion

**Figure 1:**
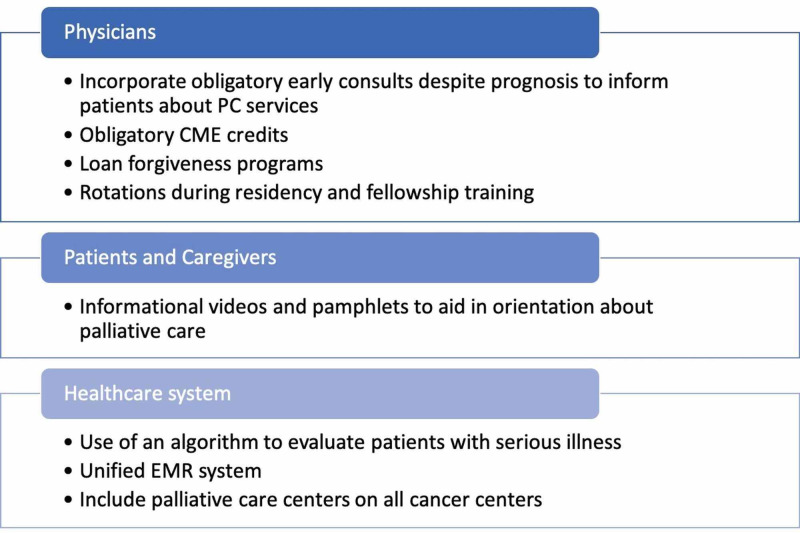
Summary of possible solutions to the three most important barriers to palliative care: physicians, family and caregivers, and the healthcare system.

Based on the literature review, we have found that there is a delay in the referral of patients with hematologic malignancies and BMT to a PC specialist. Three specific barriers to early referral have been identified: physicians, patients, and family members, and the healthcare system (Figure 1). Among physicians, patients, and caregivers, there is a misunderstanding that referral to PC implies ending their hopes or giving up [12,26]. Because of the recent emergence of PC as a distinct specialty, there is a general lack of knowledge about what the service can offer and is often confused with hospice or end-of-life care. Misconceptions about the specialty have been extensively documented among physicians, patients, and their caregivers. This is extremely important considering that in a study conducted among US adults, nearly 80% of interviewees would rely on health care providers’ information regarding PC as their most trusted source [17]. Additionally, Taber et al. mentioned that 90% of adult interviewees in New York would recommend PC to a loved one after being adequately informed of what PC comprises. Raising awareness about the specialty to physicians would eventually lead to a better understanding of PC among patients and family members regarding the multitude of services that can be offered and how they can improve the quality of life, mental health, and spiritual well-being of patients with serious illness.

The training of medicine, nursing, and allied profession’s students is of paramount importance. It is well-known that misperceptions and misrepresentations start early in training. The development of a specific PC curriculum engrained in students’ curricula is a goal that needs to be achieved. Some attempts could be tried in practice through the use of elective rotations although this approach may not work due to time constraints, personal interest among students, or limited availability of opportunities in the field. Dissemination of PC's role and function in medicine, nursing, and other medical areas should be targeted through interest groups, journal clubs, and formal curricula. Facilitation of PC rotations early in clerkships may also help this goal. A possible solution to the lack of knowledge about PC among physicians could be to incorporate this subspecialty into the curriculum of residency programs, especially in primary care specialties such as Internal Medicine, Family Medicine, and Pediatrics. Likewise, hematologist oncologists could be encouraged to get trained in PC introductory courses if they received CME credits that would count towards their recertification requirements [19]. Additionally, as previously mentioned, physicians can be incentivized to pursue fellowships in PC if such fellowships provided loan forgiveness. It is necessary to increase awareness of and training for PC as there is a niche and demand for PC specialists [10].

Furthermore, implementing a model of early referral to PC has also proven successful in improving the understanding of patients about PC. Evidence of this is a study that showed how 92% of patients informed and exposed to early referrals to PC felt better informed about the specialty and more eager to use their services in the future [27].

One way to inform patients, family members, and caretakers could be to provide educational multimedia content on a central platform, possibly through a PC advocacy organization. Taber et al. showed that 30.5% of US adults reported they would first turn to the internet and social media for information regarding PC and over 80% of the US population stated that they first searched on the internet the most recent time they were looking for information [17]. Therefore, the internet and social media are great platforms to expose patients and caregivers to accurate information from reliable resources. Ideally, patients would first be exposed to PC from their physicians and can be directed to the informational videos and pamphlets for detailed explanations on what PC encompasses. The education of caregivers is crucial to the successful delivery of PC services because research showed that 49.4% of family members and caretakers shared decisions equally with patients, were active members of the decision-making process by inquiring about prognosis and treatment, addressed collateral decisions regarding treatment choices and decisions about emergency care, and helped patients who preferred to delegate healthcare decisions to a proxy [28].

**Figure 2:**
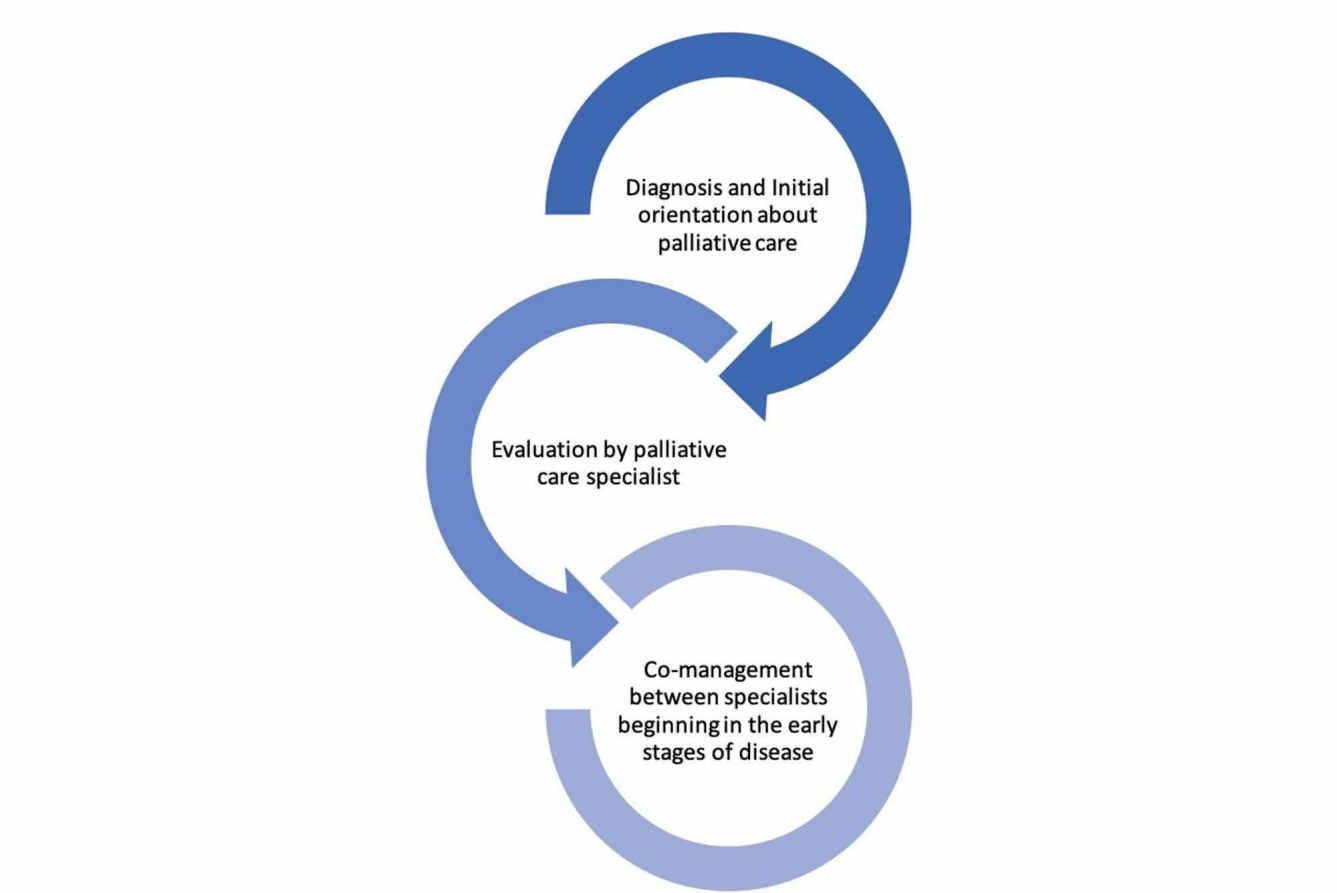
Ideally, patients should be oriented about palliative care at the time of diagnosis by their hematologist-oncologist. Understandably, a patient's emotions towards their diagnosis may interfere with their comprehension of palliative care during the initial visit so a second evaluation would be warranted to include a thorough explanation of the services palliative care offers and should also be extended to their caregivers by PC specialists. Finally, after the evaluation and referral, patients can expect a collaborative approach to their illness.

To address the issues of hematologist-oncologists struggling with when is the right time to refer patients to PC [29,30], an algorithm was proposed to determine if a BMT patient qualified for a PC consultation. The algorithm included 10 criteria for which the score needed to be more than five and included the functional status of the patient, serious post-transplant complications, significant physical or psychosocial comorbidities, other issues such as financial troubles, lack of support, uncontrolled physical symptoms, moderate to severe distress secondary to their disease or treatments, concerns about decision-making, family requests consult, prolonged hospital stays, and finally, frequent hospital readmissions since discharge [31]. This option should be explored further since the scoring system will need to be extremely sensitive in detecting patients who need the referral without excluding others who might also benefit. An easier possibility would be to require a mandatory consultation or orientation with a PC specialist at the time of the diagnosis, in which the patient can be oriented about PC and the services they provide regardless of prognosis (Figure 2). Physicians have raised a concern about how the patient’s mood, hope, or desire for treatment might suffer from early consultations with PC, but research has proven that there is no correlation between consulting PC and causing a negative impact on the patient’s overall well-being [32].

Another concern that we found among physicians is that some providers do not want to “share” or “lose” the leadership in the management of their patients [12]. Evidence showed that 40% of transplant physicians doubted the knowledge PC physicians possessed about hematologic malignancies and BMT [18]. Once there is an understanding of PC's role and physicians understand that both specialties can help in co-managing disease, hematologists, transplant physicians, and PC clinicians can approach disease in a multidisciplinary, unified manner starting the day of the diagnosis which will also help familiarize the patient and their caregivers with the whole team and avoid the interruption of care.

One of the most pressing issues that PC specialists complain about is communication. Per McCaughan et al. PC physicians, as well as primary care physicians (PCP), have mentioned how they would miss critical information regarding changes in prognosis, therapeutic treatment, and advanced directives, and how the lack of a common information platform caused a disruption in the relaying of information that would better facilitate care for patients [20]. One way to address this issue would be to create a unified electronic medical record or communication system which could be shared between PCP, PC specialists, hematologist oncologists, and transplant physicians to ensure that information can be accessed by any specialist at any given time and that will assist in the co-managing of the patient's disease. Another solution for the lack of communication could be through the nurses since they are valuable parts of the PC team. Being first responders and first-hand consultants who triage most of these patients, they support efforts clinically, educationally, and they are navigators and liaisons between providers, patients, and teams. The development of a nursing provider patient network may be crucial for early interventions.

Finally, as mentioned by LeBlanc et al., another obstacle for lack or delayed referral mentioned by physicians was the distance between clinics [16]. For this reason, including PC services in every cancer treatment center would be of vital importance. The American College of Surgeons’ Commission on Cancer established that in order to be able to receive the accreditation of Comprehensive Cancer Centers, there needs to be an available PC team on service [12]. As previously discussed, PC can help patients in many aspects, but most importantly, it will substantially improve their quality of life while also assisting physicians in the management of serious illnesses. Patients with hematologic malignancies and BMT have complicated, and unpredictable disease courses and an early referral to PC services will strongly benefit and improve their disease course by making their symptoms and hardships more manageable.

## Conclusions

As previously mentioned, PC is a relatively new specialty and the knowledge and perceptions about what it entails are full of misconceptions not only among patients but physicians too. Educating physicians about the vast amount of services PC provides to patients is key. Consensus attests to the many benefits it offers not just to patients but also to their family members and caregivers. Current guidelines recommend that patients with hematologic malignancies and BMT be referred more often and earlier to palliative care. Additional research needs to be conducted regarding possible solutions to increase the referral and accessibility of PC for BMT.
